# Minimal Detectable Bone Fracture Gaps in CT Images and Digital Three-Dimensional (3D) Radii Models

**DOI:** 10.1007/s10278-024-01185-9

**Published:** 2024-07-09

**Authors:** Martin Bittner-Frank, Andreas Strassl, Ewald Unger, Lena Hirtler, Barbara Eckhart, Markus Koenigshofer, Alexander Stoegner, Kevin Staats, Franz Kainberger, Reinhard Windhager, Francesco Moscato, Emir Benca

**Affiliations:** 1 Department of Orthopedics and Trauma Surgery, Medical University of Vienna, Währinger Gürtel 18-20, 1090 Vienna, Austria; 2 Center for Medical Physics and Biomedical Engineering, Medical University of Vienna, Vienna, Austria; 3 Department of Biomedical Imaging and Image-Guided Therapy, Medical University of Vienna, Vienna, Austria; 4 Center for Anatomy and Cell Biology, Medical University of Vienna, Vienna, Austria; 5 Ludwig Boltzmann Institute for Cardiovascular Research, Vienna, Austria; 6 Austrian Cluster for Tissue Regeneration, Vienna, Austria

**Keywords:** Computerized models, Computed tomography, Wrist fracture, Dimensional measurement accuracy, Trauma surgery

## Abstract

Knowledge of the minimal detectable bone fracture gap is essential in three-dimensional (3D) models, particularly in pre-operative planning of osteosynthesis to avoid overlooking gaps. In this study, defined incisions and bony displacements ranging from 100 to 400 µm were created in diaphyseal radii in 20 paired forearm specimens and verified with light microscopy. The specimens were scanned utilizing different computed tomography (CT) technologies/scanners, specimen positionings, scan protocols, image segmentations, and processing protocols. Inter- and intra-operator variabilities were reported as coefficient kappa. In CT images, fracture gaps of 100 µm and bone lamellae of 300 µm and 400 µm width were identified at a rate of 80 to 100%, respectively, independent of the investigated settings. In contrast, only 400µm incisions and bony displacements were visible in digital 3D models, with detection rates dependent on CT technology, image segmentation, and post-processing algorithm. 3D bone models based on state-of-the-art CT imaging can reliably visualize clinically relevant bone fracture gap sizes. However, verification of fractures to be surgically addressed should be verified with the original CT image series.

**Graphical Abstract:**

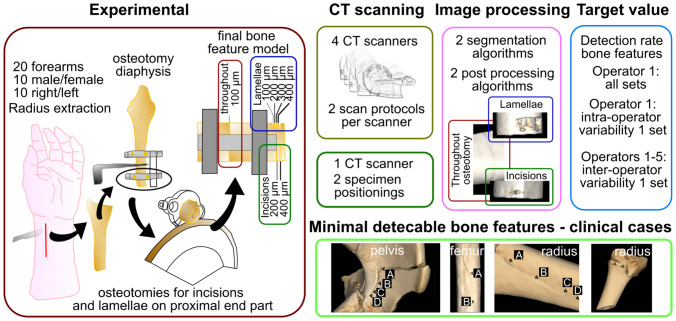

**Supplementary Information:**

The online version contains supplementary material available at 10.1007/s10278-024-01185-9.

## Introduction

Bone fractures impose a significant global burden [1] and are treated either conservatively or surgically without significant differences in the outcome, except for the distal radius [2]. The majority of bone fractures occur in the elderly [3], likely due to an increased risk of falls and decreasing bone quality [4], often necessitating surgical intervention for internal stabilization and early mobilization.

Pre-operative planning is conventionally performed using 2D radiography. In complex and articular fractures, 3D imaging, such as computed tomography (CT), is used to visualize fracture fragments, although identification of fracture lines still relies on the corresponding 2D image slices. 3D models are utilized to gain a better 3D impression of fracture parts for surgical planning, such as reduction. Recently, the use of digital 3D and additively manufactured models has gained interest [5], e.g., for surgical treatment of distal radius [6, 7], scaphoid [8], tibia plateau [9], and acetabulum [10–12] fractures.

To avoid missing fracture gaps in 3D printed models, bone fragments are often segmented individually, a time-consuming process that requires a thorough communication between radiologists, engineers, and surgeons [13]. Alternatively, processing the entire bone at once could be faster but may result in overlooked gaps in fractures requiring surgical reduction.

The goal of the current study was to determine if 3D CT-based bone fracture models, obtained with standard clinical protocols and minimal image processing, can be used for pre-operative planning without risking overlooking clinically relevant fracture gaps. In an animal model, the threshold for successful healing of a non-union was determined as 0.5 mm [14], which is thus, the minimally required detectable feature size. Other factors, such as CT scanner/technology, specimen positioning direction, scan protocol, image segmentation, and image post-processing might influence the final model accuracy and were addressed in this study.

## Materials and Methods

**Fig. 1:**
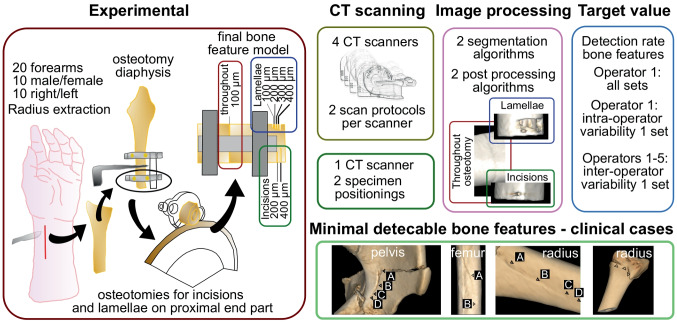
Flow-chart: Experimental preparation of bone features (throughout osteotomy of diaphysis, bone incisions, and bony displacements: lamellae). CT scanning with 4 scanners and 2 scan protocols. Additionally, 2 specimen positionings were investigated with one scanner. Image processing was done with 2 segmentation protocols and 2 post-processing algorithms. Target value was the detection rate of defined bone features. Further, inter- and intra-operator variability were assessed. Lastly, verification of detection of minimal bone feature sizes was performed in CT image series and 3D models in clinical cases (marked fracture line with triangles and labeled from A–D, see Supplementary for more information)

Conduction of this pilot study on anatomic forearm specimens, as well as using retrospective clinical data, was approved by the Ethics Committee of the Medical University of Vienna (EK-Nr: 2003/2019 and EK-Nr: 1284/2019, retrospectively). Figure 1 illustrates the study flow, highlighting the most important steps.

### Anatomic Forearm Specimens

Paired human forearm specimens (left and right) were obtained from 10 body donors (5 male and 5 female, average age 78 ± 8 years) from the Division of Anatomy, Center for Anatomy and Cell Biology, Medical University of Vienna, and stored individually in airtight plastic containers at − 20 °C.

### Fracture Preparation and Verification

**Fig. 2:**
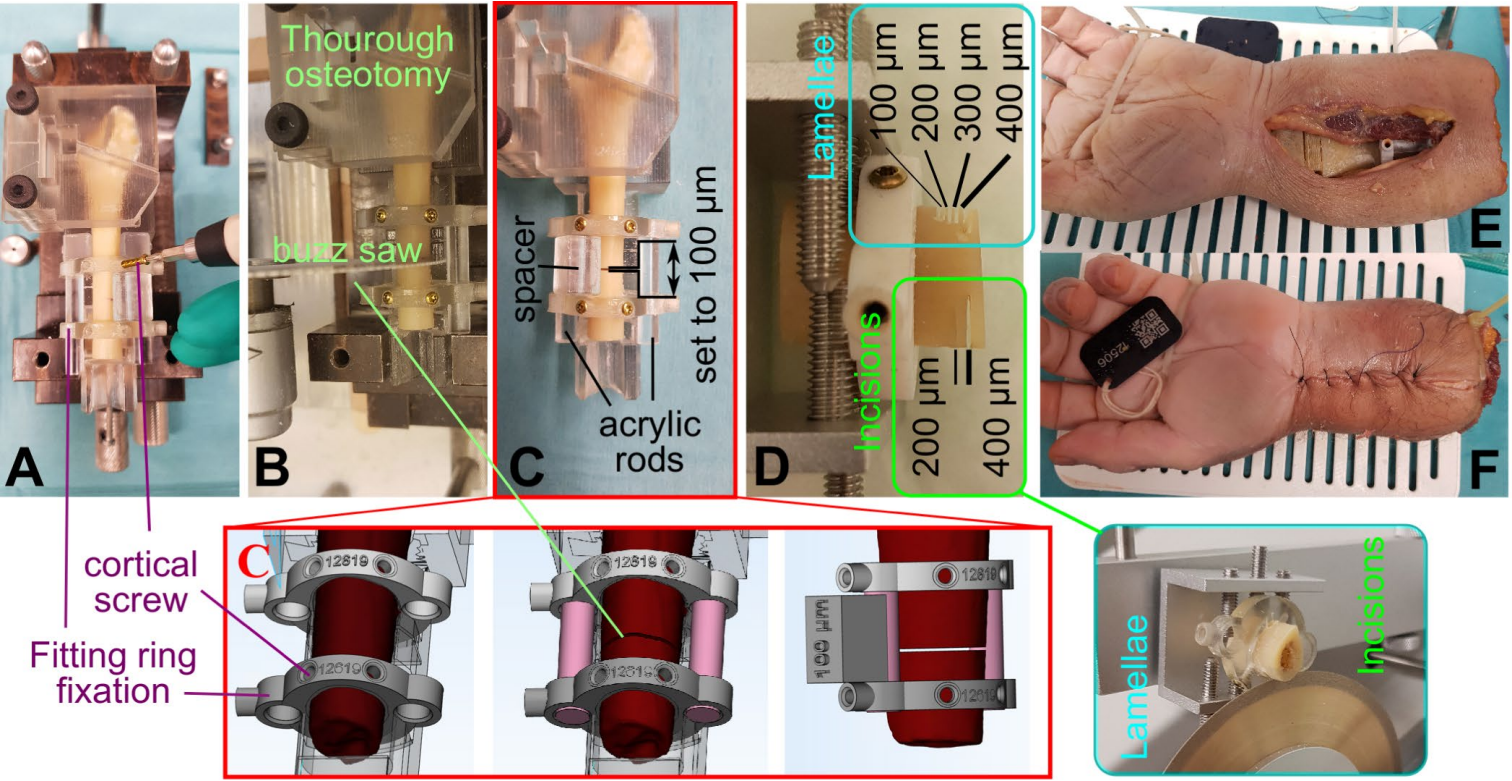
Fracture preparation: **A** Fixation of distal and proximal additively manufactured fitting rings to the diaphysis using cortical screws. **B** Throughout osteotomy with buzz saw. **C** Adjustable fracture gap via spacer and acrylic rods. Inset: Design and concept of throughout osteotomy with fitting rings and spacer. **D** Precision cuts to create bony displacements (lamellae, cyan) and incisions (green); see inset in the right bottom. **E** Re-implantation of fractured radii into forearm specimens. **F** Wound closure with subcutaneous and cutaneous suturing

Extraction of the radii was performed using a modified Henry approach, removing all attaching soft tissue. The explanted radii were macerated in water at 60 °C for 14 days removing the periost and residual soft tissue. Specimen-specific additively manufactured osteotomy guides were designed in Mimics Research (V21.0 Materialise NV, Leuven, Belgium) and used to generate a throughout diaphyseal fracture with adjustable gap width (see Fig. 2A). In a parallel study, a similar approach was used to simulate a Colles’ fracture and to determine the effect of image acquisition and image processing onto the accuracy of 3D bone fracture models [15]. In the current study, high-resolution 3D surface scans of all macerated bones were performed with an optical scanning system (SmartSCAN HE-C8-8MP, Hexagon AB, Stockholm, Sweden; feature accuracy of 14 µm). These scans were used as negatives to design almost perfect fitting osteotomy guides and fitting rings. 3D printing was performed with a PolyJet printer (Connex 3 – Objet 500, Stratasys, Rechovot, Israel; layer thickness, 16 µm; accuracy, up to 200 µm for the entire model). The diaphysis was osteotomized in between the rings using a buzz saw (see Fig. 2B). Two acrylic rods linked the two rings, and a defined fracture gap size was adjusted using a spacer (see Fig. 2C). Additionally, a low-speed precision cutter (IsoMet, Buehler ITW Test & Measurement GmbH, Leinfelden-Echterdingen, Germany) was used to generate bone incisions and bone lamellae (ssee Fig. 2D) with a 400-µm-wide blade. The osteotomy and generation of bone lamellae were repeated with a 200-µm-wide blade, to determine the potential effect of the blade width on feature detectability. The size of created incisions, bone lamellae, and the fracture gap (determined at margo interosseus and posterior) was performed with a microscope (Zeiss Axio Imager, Carl Zeiss AG, Oberkochen, Germany). Finally, the fractured radii were re-implanted into the forearm specimen (see Fig. 2E, F) to allow for realistic CT scanning conditions, accounting for the influence of soft tissue presence.

### Imaging Protocols

CT imaging was performed with three different state-of-the-art energy-integrating detector (EID) CT scanners: SOMATOM Force, SOMATOM Edge Plus (both Siemens Healthineers AG, Forchheim, Germany), and Diamond Select Brilliance64 CT (Koninklijke Philips N.V., Amsterdam, Netherlands). Further, a photon-counting detector (PCD) CT (NAEOTOM Alpha, Siemens Healthineers AG, Forchheim, Germany) was used. The routine scan protocol (according to in-house clinical standards for suspected wrist fractures) of each scanner as well as an optimized scan protocol were used. “Optimized” in this context refers to increased image quality (increased tube current time product and decreased pitch factor) to demonstrate the feasibility of CT in fracture imaging regardless of patient’s radiation exposure. The parameters (see Table 1) were designed for each scanner aiming to allow for uniform protocols and an inter-device comparison. This process was limited due to the usage of different scanner generations, manufacturers, and therefore properties. Further, a trade-off had to be made due to different restrictions of the x-ray tube systems to examine all specimens in a reasonable period with high x-ray generator output. All scans were performed with the forearm specimens positioned in their longitudinal direction, and additionally at 90° rotation for the SOMATOM Force.

**Table 1:** CT scan protocols and reconstruction settings

**Scanner**	**Alpha**		**Force**		**Edge+ **		**Brilliance**	
**Scan technology**	**PCD**		**EID**		**EID**		**EID**	
**Scan protocol**	**Optimized**	**Routine**	**Optimized**	**Routine**	**Optimized**	**Routine**	**Optimized**	**Routine**
**Scan settings**								
Collimation in mm	120 × 0.2	120 × 0.2	64 × 0.6	64 × 0.6	64 × 0.6	64 × 0.6	64 × 0.625	64 × 0.625
Voltage in kVp	120	120	120	120	120	120	120	120
mAs	250	55	350	76	350	85	300	80
Rotation time in s	0.5	0.5	1	1	1	1	1	1
Pitch factor	0.80	0.80	0.20	0.85	0.35	0.85	0.20	0.39
Mean CTDI in mGy	20.1	4.4	19.9	4.4	23.7	5.8	26.5	5.3
Mean DLP in mGycm	230	50	490	114	594	152	668	150
Matrix size	512 × 512							
**Reconstruction settings**								
Slice thickness in mm	0.20	0.20	0.40	0.40	0.50	0.50	0.67	0.67
Increment in mm	0.20	0.20	0.40	0.40	0.50	0.50	0.67	0.67

*EID* energy-integrating detector, *PCD* photon-counting detector, *CTDI* computed tomography dose index, *DLP* dose length product

### Image Processing and Fracture Identification

**Fig. 3:**
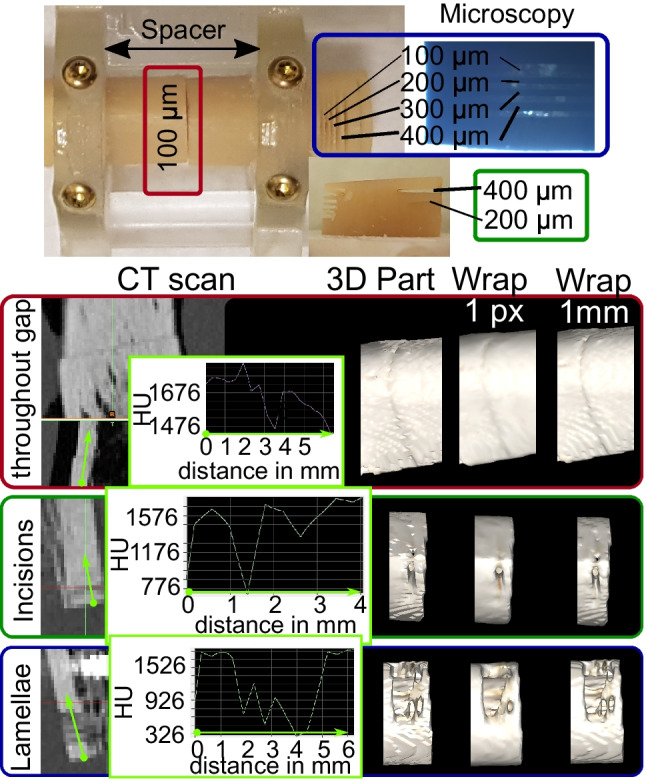
Osteotomies for manufacturing throughout complete fracture gap (red), incisions (green), and bone lamellae (bony displacements, blue). Detection rate was assessed in CT images (for illustration purposes, a line intensity profile is shown) and in digital 3D models at the displayed processing steps

CT image series were processed and segmented in Mimics Research (V21.0, Materialise NV, Leuven, Belgium) to obtain digital 3D radii models. This is one of the most widely used software for medical image processing in additive manufacturing and one out of only three software that is available with a medical license [16]. Image segmentation was performed with an automatic threshold (AT) larger than 226 Hounsfield Units (HU) for bone (CT), which is predefined in Mimics as the standard threshold for bone. Then, a 3D part was generated (setting “optimal”), and post-processed with the “wrap” (smallest detail, 1 pixel) and “smooth” tool (smooth factor, 0.3; 2 iterations, as described previously [17]). Hereby, wrap inflates the surface to close small gaps and holes, whereas smooth removes sharp edges, which is a standard procedure, as also suggested in segmentation tutorials from Materialise. Different mesh generations (e.g., with lower number of triangles) were not investigated, since Gelaude et al. [17] have already demonstrated the superiority of these settings, and computation time and cost were already minimal. Additionally, a wrapping distance of 0.1 mm was selected. Further, scans obtained with the optimal protocol of the SOMATOM Force were segmented with a manually determined threshold (MT), based on a line intensity profile and determination of the 50% difference between soft tissue and the cortex, as described previously [18]. Fracture identification was performed initially by a single operator (OP 1) in a binary manner (discontinuity in the cortex visible or not visible), as exemplarily illustrated in Fig. 3, and verified by a senior radiologist, specialized in musculoskeletal radiology. This procedure was repeated after 8 weeks to determine the intra-operator variability. Additionally, inter-operator variability was determined for the variables in CT images from the SOMATOM Force and corresponding 3D models, evaluated by four additional operators (OP 2–5). OP 1 and 2 were biomedical engineers with several years of experience in image processing. OP 3 was a biomedical engineer and OP 4 a medical fellow, both with some experience in image processing. OP 5 had no image-processing experience.

### Statistical Analysis

Statistical analysis of the detection rates was performed in SPSS (v27, IBM Corporation, Armonk, NY, USA) using a related-samples Chochran’s *Q* test and a significance level of 0.05, adjusted by the Bonferroni correction for multiple tests. Inter- and intra-operator variabilities were analyzed in terms of coefficient kappa. Since the binary data were non-normally distributed across samples (either a feature was detectable in almost all samples, or in none), the hypothetical probability of chance agreement was adjusted according to Brennan and Prediger [19] for Cohen’s kappa as $\frac{1}{z}$ (*z*, 2 categories) and to von Eye [20] for Fleiss’ kappa as $\frac{1}{1-{z}^{d}}$ (*d*, 5 raters).

## Results

### Microscopy

All incisions were generated with the precision cutter (to create defined bony displacements (lamellae of remaining bone) and incisions (removed bone)) and could be successfully evaluated (except for two incisions on the initial sample used for adjustments). In contrast, three out of 20 complete fracture gaps (those set to 100 µm) could not be evaluated due to relative displacement of fracture parts and their subsequent tilting during re-implantation. Verification of incisions created by the precision cutter indicated an average relative deviation of 9.8% for bone lamellae, 0.5% for incisions, and 38% for the 100 µm throughout diaphyseal osteotomy (for absolute values, see Table 2).

**Table 2:** Verification of fracture gaps with microscopy

**Measurement**	**Lamellar width in µm**				**Incision width in µm**		**Fracture gap**	
	**400**	**300**	**200**	**100**	**400**	**200**	**m.io**	**m.p.**
	**400-µm blade**						**700-µm blade**	
Mean width in µm	416	310	210	127	404	200	153	122
SD width in µm	18	12	12	18	5	6	77	44
	**200-µm blade**						**200-µm blade**	
Mean width in µm	439	316	229	135				107
SD width in µm	30	18	20	23				26

*m.io* margo interosseus, *m.p.* margo posterior

### Effect of Scan Technology, Scan Device, and Scan Protocol in CT Images and Digital 3D Models

**Fig. 4:**
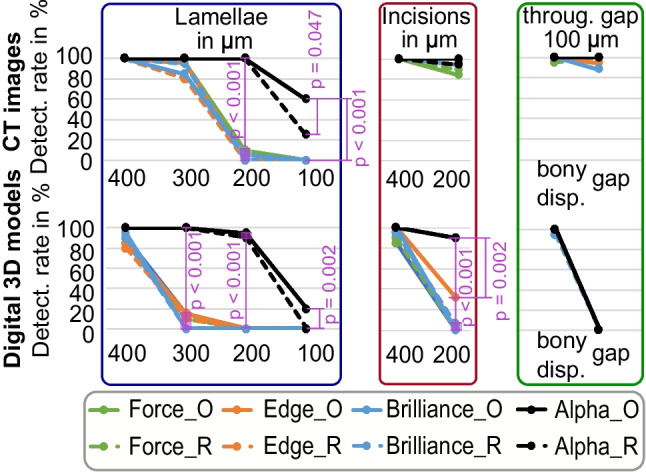
Fracture detection rate in CT images (top) and corresponding digital 3D models (bottom). O, optimized protocol; R, routine protocol; disp., displacement. Significant differences are marked with bars, and corresponding *p*-values are provided (adjusted with Bonferroni correction for multiple testing)

In PCD-CT image series, bone lamellae of 100 and 200 µm indicated significantly higher detection rates (*p* < 0.001, see Fig. 4, top) than EID-CT images. Similarly, in 3D models, bone lamellae of 100, 200, and 300 µm width as well as 200-µm incisions were detected at a similarly high rate (see Fig. 4, bottom). Further, the optimized scanning protocol for PCD-CT images and 3D models was superior to the routine protocol for the detection of 100-µm bone lamellae (*p* = 0.047).

Interestingly, there was no effect of CT scanner or scan protocol for EID-CT image series or corresponding 3D models. The detection rate in CT images for the fracture gaps/incisions (100, 200, and 400 µm) and the 300- and 400-µm bone lamellae was in the range between 84 and 100% (see Fig. 4, top). Bone lamellae of 200 µm width could be detected in only 5 to 10% of specimens, and 100-µm lamellae were not detectable at all. The throughout fracture of the diaphysis (set to 100 µm) indicated a bony displacement, detectable in 95 to 100% of samples. In 3D models, the detection rate for the 400-µm bone lamellae was in the range between 80 and 95% and 10 and 15% for the 300-µm lamellae (see Fig. 4, bottom). The 200- and 100-µm lamellae were not detectable. The 400-µm incisions were visible in 80 to 100% and 200-µm incisions in 0 to 32% of cases. The complete 100-µm gap was not detectable at all, but the bony displacement was visible in 80 to 100% of samples.

### Effect of Segmentation and Image Processing in Digital 3D Models

**Fig. 5:**
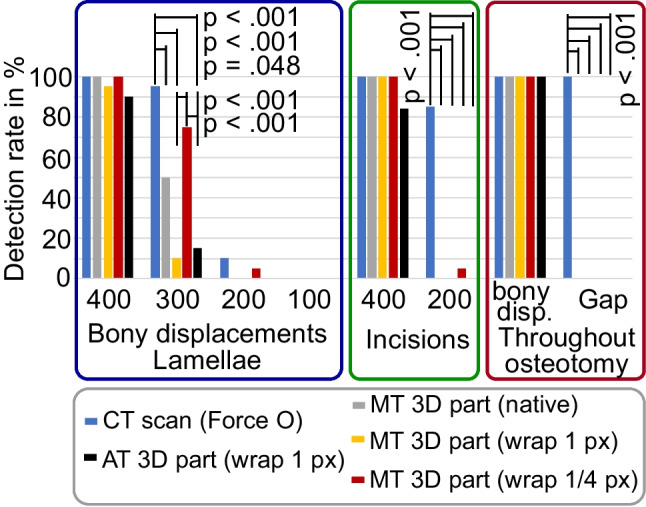
Fracture detection rate for CT images obtained with the SOMATOM Force (optimized protocol), corresponding digital 3D models segmented with an automatic threshold (AT) and manual threshold (MT). Significant differences are marked with bars, and corresponding *p*-values are provided (adjusted with Bonferroni correction for multiple testing)

The detection rates for the 300-µm bone lamellae and the 100- and 200-µm fracture gaps were significantly lower in segmented digital 3D models than in corresponding CT image series (*p* < 0.001; see Fig. 5). Post-processing of the digital 3D models (wrapping and smoothing) decreased the detection rate further from 50 to 10% for the 300-µm lamellae. However, this effect did not demonstrate statistical significance (*p* = 0.122). In contrast, selection of a wrapping distance of 1/4 pixel instead of 1 pixel increased the detection rate significantly from 10 to 75% (*p* < 0.001). There was no significant difference in the detection rate between the digital 3D models segmented using the automatic and the manually determined threshold method, for any investigated fracture gap or bone lamellae size.

### Effect of Specimen Positioning and Spacing Between Bony Displacements

**Fig. 6:**
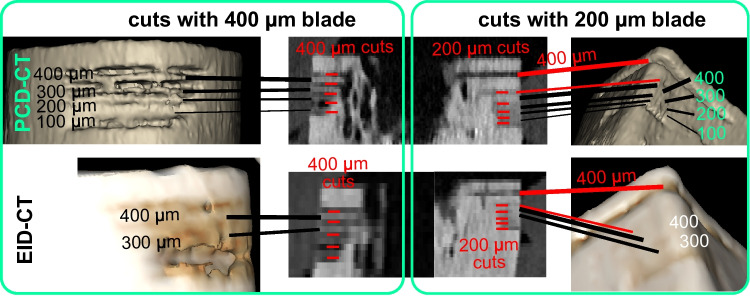
3D models generated from PCD (top) and EID (bottom) CT image series, showing bony displacements (lamellae) generated with a blade width of 400 µm (left) and 200 µm (right). Additionally, a single incision with 400 µm, separated by 1 mm, is shown on the right

Specimen positioning did not influence the detection rate for any investigated bone feature. In contrast, the spacing between bony displacements (400 µm vs. 200 µm) for bone lamellae and a performed throughout osteotomy with a 200-µm blade instead the 700 µm blade) had a significant effect on bone feature detectability (see Fig. 6). A detailed description of the results is provided in the Supplementary.

### Intra- and Inter-operator Variability

The intra-operator variability indicated an almost perfect agreement (> 0.80), except for the 200-µm bone lamellae in CT images and 300-µm bone lamellae in 3D models (see Table 3). Similarly, the inter-operator variability showed a substantial (> 0.60) to an almost perfect agreement (> 0.80), except for the 200-µm bone lamellae in CT images, and the 400-µm bone lamellae in post-processed 3D models (see Table 4).

**Table 3:** Intra-operator variability (for operator 1) reported as Cohen’s kappa, adapted according to [19]

	**Lamellar width in µm**				**Incision width in µm**		**Fracture gap**	
	**400**	**300**	**200**	**100**	**400**	**200**	**Step**	**Gap**
CT Force optimal	1.00	1.00	0.50	1.00	1.00	0.68	1.00	1.00
3D Part AT	1.00	0.10	1.00	1.00	0.89	0.89	1.00	1.00
Wrap 1 px	0.90	0.90	1.00	1.00	0.89	1.00	1.00	1.00

**Table 4:** Inter-operator variability (for all 5 operators) reported as Fleiss’ kappa, adapted according to [20]

	**Lamellar width in µm**				**Incision width in µm**		**Fracture gap**	
	**400**	**300**	**200**	**100**	**400**	**200**	**Step**	**Gap**
CT Force optimal	0.98	0.90	0.47	0.82	1.00	0.78	0.97	0.75
3D Part AT	0.80	0.65	1.00	1.00	0.87	0.78	0.93	1.00
Wrap 1 px	0.50	0.94	1.00	1.00	0.79	0.88	0.94	1.00

### Clinical Examples in Patients

Verification of the determined minimal bone feature size in this pre-clinical study was performed with CT image data and corresponding 3D models of actual clinical cases at the University Hospital Vienna. A detailed description with images describing the observed fracture, its run, and size is provided in the Supplementary material. In principle, there was a relevant discrepancy between detectability in CT image series and corresponding 3D models. Fracture gaps below approximately 0.5 mm were mostly not detectable in 3D models, although they were clearly visible in CT image series.

## Discussion

The present study determined the minimal detectable bone feature size in CT image series and corresponding digital 3D bone models. Detection of fracture gaps in CT images was feasible down to 100 µm, indicating sub-pixel accuracy (resolution 0.40 to 0.67 mm in EID and 0.078 mm in PCD-CT images). This sub-pixel accuracy can be attributed to the partial volume effect (PVE). In gaps smaller than the image resolution, the HU value of voxels containing the gap is a composite of the HU values of bone and soft tissue (an average of “pure” bone and soft tissue). Therefore, they are distinguishable from voxels of actual cortical bone [18]. Previously, it was determined that the minimum feature accuracy is limited by the scan resolution, according to the sampling theorem [21]. However, using proper CT segmentation and 3D reconstruction, other studies also generated bone models at sub-voxel accuracy [22, 23].

The detection rate of bony displacements was dependent on scan technology and spacing in between. In accordance, PCD-CT images have previously been reported to be superior to EID-CT images for visualizing small bone features [24–26]. Interestingly, in the present study, the detection rate was independent of the chosen EID-CT scanner, scan protocol, or specimen positioning. In EID-CT-based 3D models, fracture gaps and bone lamellae were only detectable at a width of 400 µm. Thus, segmentation and subsequent image processing caused a substantial loss of feature accuracy. Selecting a smaller feature detail in wrapping increased the detection rate for the 300-µm bone lamellae but caused an increased surface roughness of the digital 3D models. Although 100-µm gaps were not directly detectable in digital 3D models, a bony displacement (fracture dislocation along the fracture surface) was detectable in 80 to 100% of cases. A retrospective analysis of clinical cases demonstrated that even minimal dislocations (~ 0.5 mm) of bone fragments were detectable in digital 3D models as dislocations in the cortex, but not as a gap. Smaller fracture gaps (< 0.5 mm) were not detectable in 3D models and led to an underestimation of the fracture line length.

To the best of the authors’ knowledge, the determination of the minimal detectable bone feature size has not been performed so far, limiting a comparison of the presented results with existing literature. Conventionally, the spatial resolution of CT image series is assessed using phantoms, e.g., the American College of Radiology CT accreditation phantom [27]. This involves separating a specific number of aluminum bars into 4 to 12 line pairs per centimeter (lp/cm), with the smallest features ranging from 0.417 to 1.25 mm.

If this is put in relation to conventional wrist protocols with a slice thickness of 0.625 mm, the protocol [28] yields a relative feature accuracy of 1 to 2 voxels. This minimal feature accuracy was exceeded in the present study, as features with 0.1-mm thickness (corresponding 1/4 to 1/6 pixel) were detectable in CT images. However, several scanning-related variables, such as the size and spacing of the detector measurements, the field of view, matrix size, and the selection of the reconstruction kernel, substantially effect the spatial resolution in CT images [29]. For example, Kakinuma et al. [30] reported that slits of 0.3 and 0.12 mm width were detectable using an ultra-high-resolution scanner (x–y resolution, 0.25 mm), but not using a conventional high-resolution scanner (x–y resolution, 0.35 mm). Hence, only a direct evaluation of features with a known physical size in reconstructed image series allows for determination of the detectable minimal feature size.

In the present study, the inter- and intra-operator reliability indicated an almost perfect agreement. Lower agreements were observed for the 200-µm bone lamellae in EID-CT images, 300-µm lamellae in 3D models, and 400-µm bone lamellae in post-processed 3D models. Hence, these values can be regarded as the uncertainty onset in minimum feature detectability. As a limitation, no direct evaluation of fracture detectability was performed on actual additively manufactured models. Depending on the selected technology, the average error of 3D printing is below 100 µm [31], meaning that no major deviations are expected between digital 3D and printed models, at least for modern high-end PolyJet devices. Another limitation was that the complete 100-µm fracture gap showed a higher deviation than the partial incisions with 200- and 400-µm width, related to the movement of fracture parts during re-implantation.

In future work, defined bone fracture gaps with known dimensions should also be 3D printed to determine the minimal detectable bone fracture gaps in real 3D models. Of course, this will also depend on the chosen manufacturing technology [32]. In clinical settings, it would be interesting to assess fracture gap sizes in 3D virtual models and further measure the same gaps intra-operatively, e.g., with a micrometer, to validate the size of observed bone gaps in virtual 3D bone models.

## Conclusion

Bone features were detectable at sub-pixel accuracy (< 400 µm), both in CT images and in corresponding digital 3D models. There was a significant effect of image post-processing, whereas EID-CT scanning showed no effect on bone feature detectability. Hence, in clinics, verification of fracture gaps present in the 3D model has to be performed with the original CT image series, ideally by a radiologist. All investigated parameters yielded minimal detectable bone features < 0.5 mm, meaning that 3D models obtained with clinical routine CT protocols and simple image processing can be used to visualize clinically relevant bone fracture gaps.

## Supplementary Information

Below is the link to the electronic supplementary material.

Supplementary file1 (DOCX 6521 KB)

## Data Availability

Data and statistical analysis is available at 10.17632/fycj3648bh.1.

## Abbreviations

3D: Three-dimensional
PVE: Partial volume effect
AT: Automatic threshold
MT: Manual threshold
OP: Operator
PCD: Photon-counting detector
EID: Energy-integrating detector
