# Supplementary material for: Minimal Detectable Bone Fracture Gaps in CT Images and Digital Three-Dimensional (3D) Radii Models
Source: J Imaging Inform Med. 2024 Jul 9;38(1):84–93. doi: 10.1007/s10278-024-01185-9 (PMC11811335; doi:10.1007/s10278-024-01185-9)
Supplement: Supplementary file 1 — Supplementary file1 (DOCX 6521 KB) [file 10278_2024_1185_MOESM1_ESM.docx]

## Supplementary material

**Statistical analysis – Sample size**

A power analysis was performed, according to Kadam and Bhalerao (18) as: $n= \frac{{{(Z}_{\alpha/2}+Z_{1-\beta})}^{2}2\sigma^{2}}{{{(\mu}_{1}-\mu_{2})}^{2}},$(using significance level (α) of 95% (type I error: 0.05) and a power (β) of 80% (type II error: 0.20), Z_α/2_: standard normal Z value, 1.96 for α = 0.05; Z_1 − β_: standard normal Z value, 0.84 for β = 80%; σ: pooled SD; μ1 – μ2: difference of means). Assuming a pooled standard deviation of 20% and difference of means of 40% (between different bone feature sizes, e.g. 200 and 400 µm incisions, both based on pilot experiments) the sample size per group was calculated as 3.9. To account for the variation in anatomy, bone density, etc., that might have acted as confounding variables, the sample size was maximized to the highest available number of anatomic specimens, namely 20.

**Effect of specimen positioning and spacing between bony displacements**

In EID-CT images lamellae of 300 µm width were less detectable (58 % vs. 95 %, p < 0.001) with smaller spacing, whereas no effect was observed for the throughout osteotomy. Similarly, in corresponding 3D models bone lamellae of 400 µm width, separated by 200 µm, were only detected in 58 % of cases, compared to 90 % for a separation of 400 µm (p = 0.07). Detection of a bony displacement in the throughout osteotomy was not affected by blade width used for cutting. In PCD-CT images, 100 and 200 µm bone lamellae were significantly less detectable, if spacing was reduced to 200 µm, instead of 400 µm. The same effect was observed in corresponding 3D models for bone lamellae of 300 and 200 µm.

**Clinical Cases**

**Obtaining CT Image data**

CT image series were retrospectively obtained from the Picture Archiving and Communication System (PACS) of the General Hospital Vienna. Inclusion criteria were a closed fracture of the distal radius, age above 18 and below 45 years (to exclude osteoporotic fractures) for the year 2019. In total, 18 patients ranging from 21 to 45 years old were included (13 males, 5 females, mean age 34 years). Additionally, a PACS search was performed by an orthopedic and trauma surgeon for patients who suffered bone fractures that required surgical treatment. As such, several topic-related clinical cases were included for analysis.

**Image processing**

Image processing was performed in the same manner as described in the methods section of the manuscript. In short, CT image series were segmented for a threshold larger than 226 HU, followed by minimal post-processing by wrapping (smallest detail: 1 px, gap closing distance: 1/2 px) and smoothing (smooth factor: .3 and 2 iterations).

**Identification of bone features in clinical CT images and corresponding 3D models**

Figure S 1 to Figure S 6 illustrate selected clinical examples, whereby the fracture gap distance along the fracture line is decreasing. Although the progression of the fracture lines can be properly seen in the CT image series, they are only partially presented in the corresponding digital 3D model. Hence, these 3D models can be used to sufficiently display only large (> .5 mm) gaps.

Additionally, Figure S 7 demonstrates a bony displacement of ~.5 mm size, which can be properly identified in the CT image series and corresponding 3D model. In accordance with the outcome of the pre-clinical study described in the manuscript, the size of displacements necessary for the identification, is likely dependent on the adjacently tissue, e.g. bone vs. soft tissue. As such, small bony displacements are visible at sub-voxel resolution, if they are surrounded by soft tissue, due to the PVE.


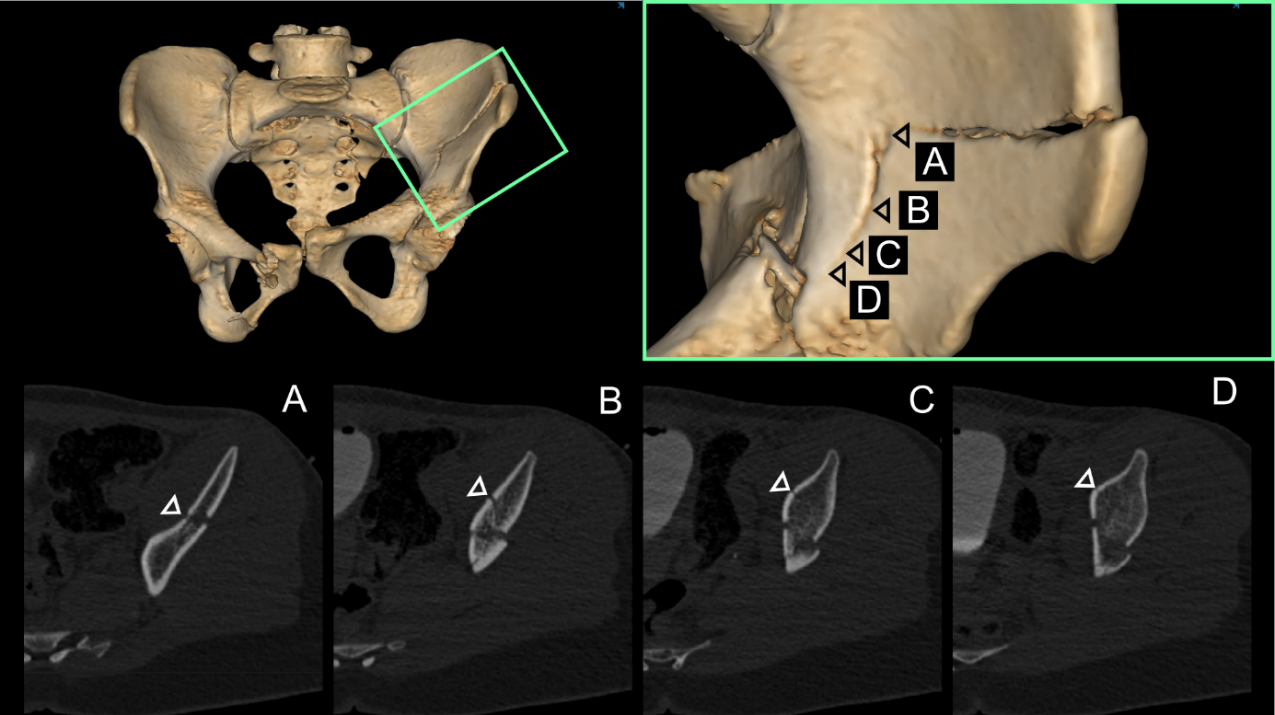


Figure S 1: Digital 3D model of a complex pelvic fracture (female, 26 years old) created from a CT imaging series (scanned at 120 kV, slice thickness: .75 mm, increment: .5 mm; reconstruction kernel: B20s). The fracture line starts at the iliac wing and appears to continue into the ischium, which is indicated by the displacement of the ischium and follows the rules of stress transmission by the initial trauma. However, the 3D model does not provide a continuously visible fracture line (A-C: visible, D: not visible, likely due to the decreasing fracture gap size). Corresponding CT images are provided for each indicated position in the 3D model.


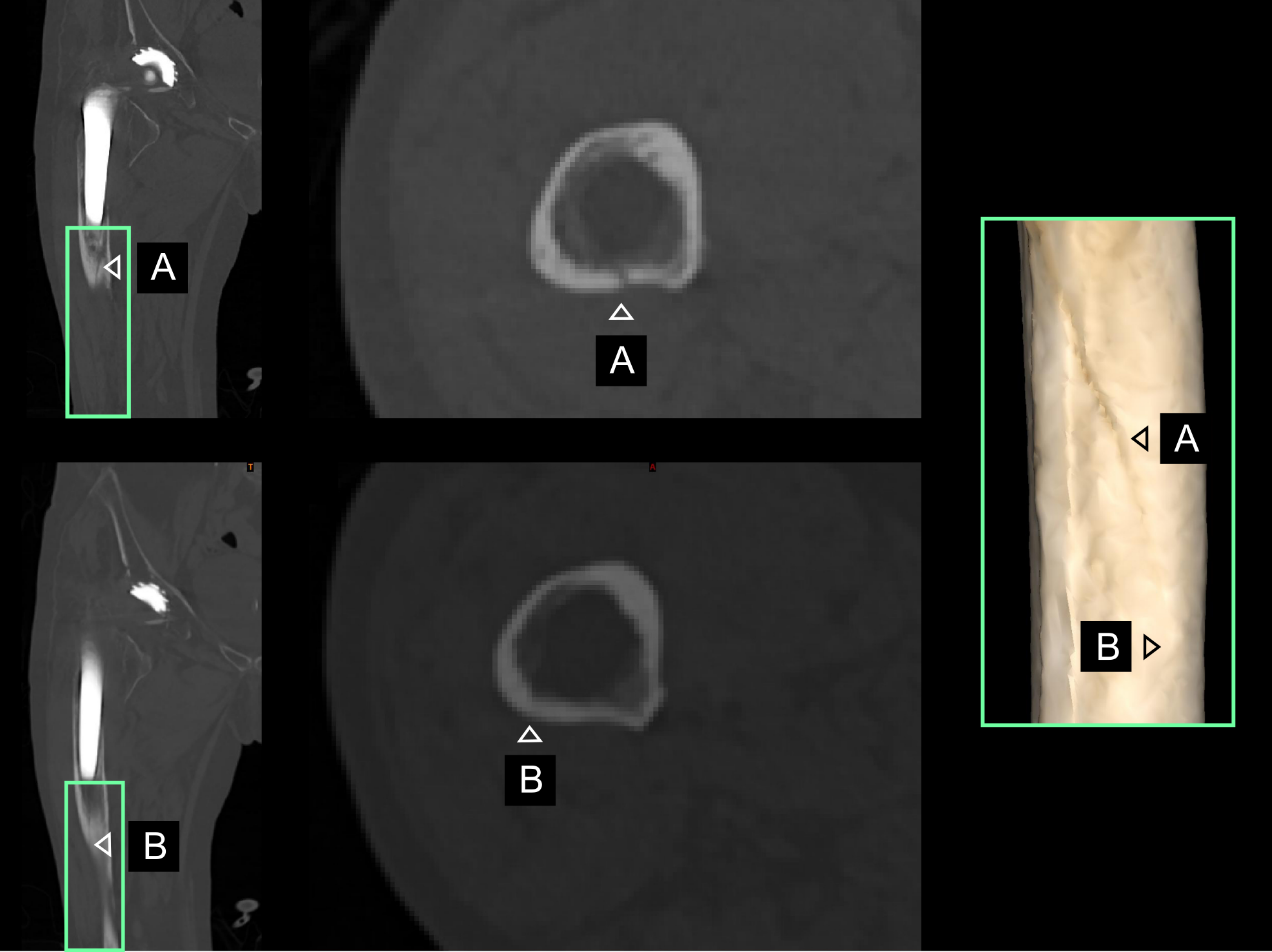


Figure S 2: CT images (scanned at 120 kV, slice thickness: 1.8 mm, increment: .8 mm; reconstruction kernel: Br38s\3) of a traumatic intertrochanteric periprosthetic fracture (right side) extending obliquely in inferior direction (female, 90 years old). A: oblique fracture line visible in the CT images and visible as displacement in the 3D model. B: The most inferior portion of the fracture line is visible in CT images, but not in the 3D model. Overall, the fracture line in the 3D model is not visible in the most inferior 26 mm, compared to the CT image series.


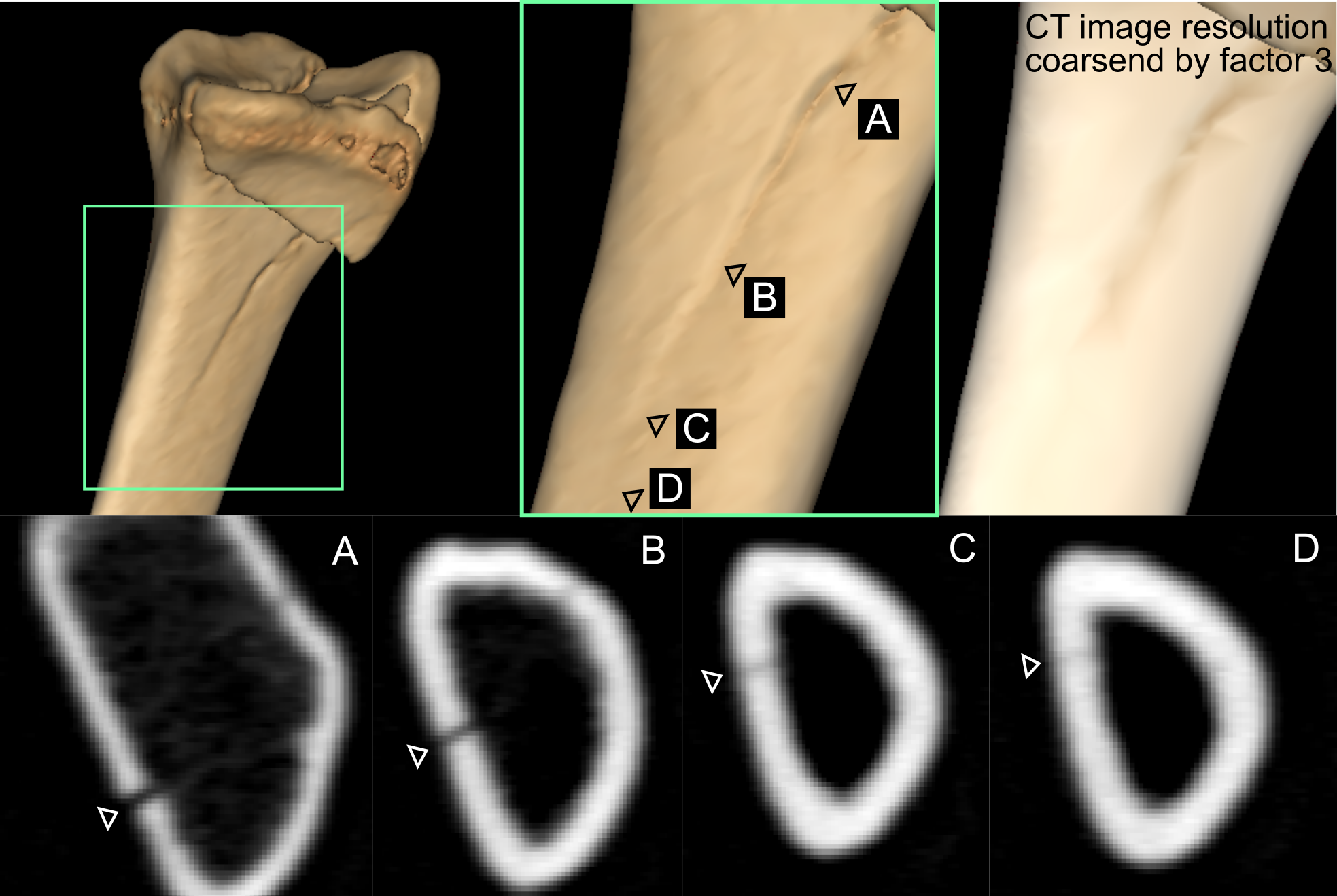


Figure S 3: Digital 3D model of a distal radius fracture with fracture line extending into the diaphysis (male, 28 years old) created from a CT imaging series (scanned at 120 kV, CT pixel size: .25 mm, slice thickness: .6 mm, increment: .3 mm, reconstruction kernel: B31s; bottom). A: Clear fracture gap in CT image and model. B & C: Gap of .7 mm and .4 mm clearly visible in 3D model. D: Gap of .3 mm not visible in the 3D model (fracture line ends 4 mm earlier than in CT image series). Additionally, the top right 3D model is generated from the same image series, whereby the image resolution was coarsened by a factor of 3 (pixel size .76 mm, increment .9 mm). Hereby, the fracture line ends more distal than in the un-coarsened model.


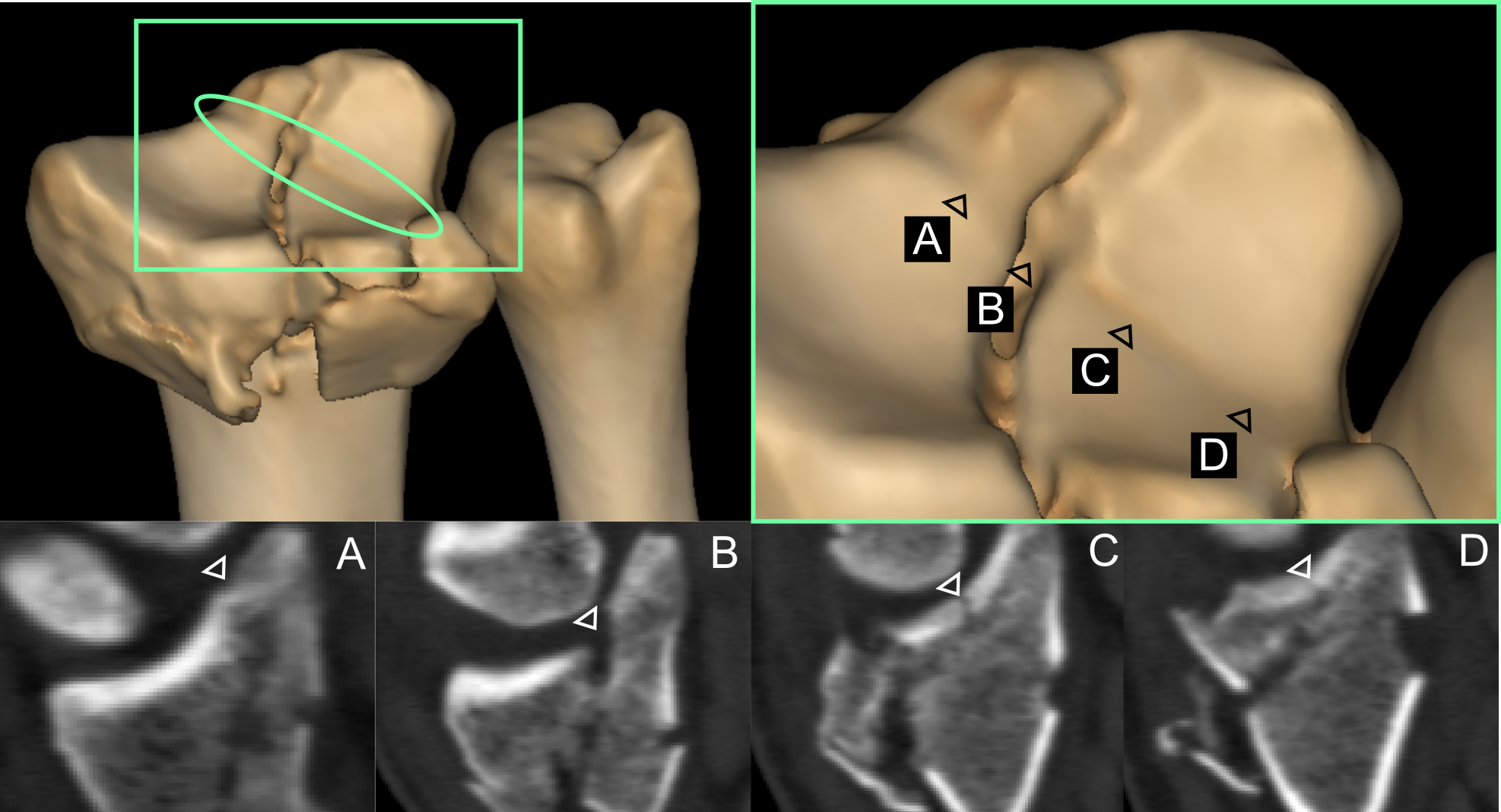


Figure S 4: Digital 3D model of a complex articular radius fracture (male, 30 years old) created from a CT imaging series (scanned at 120 kV, CT pixel size: .30 mm, slice thickness: .6 mm, increment: .3 mm, reconstruction kernel B31s). A: Discontinuity in CT image and model. B: Gap of 1.6 mm clearly visible in 3D model. C: Gap of .5mm at the osteo-articular surface still visible as mold in 3D model. D: Decreased gap size, not visible in the 3D model.


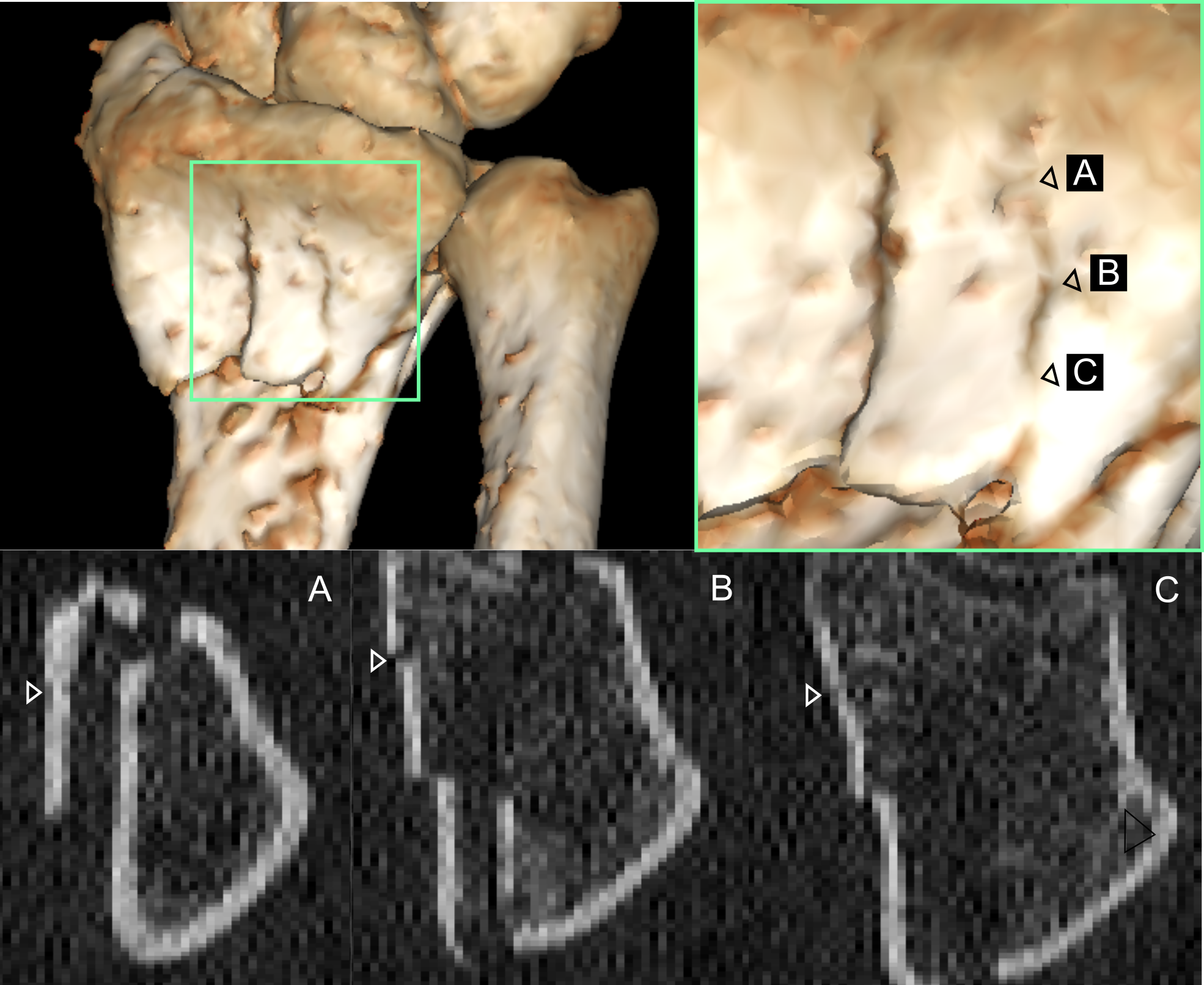


Figure S 5: Digital 3D model of a complex distal radius fracture (male, 30 years old) created from a CT imaging series (scanned at 120 kV, CT pixel size: .57 mm, slice thickness: .6 mm, increment: .3 mm, reconstruction kernel Ur77u). A: Discontinuity in CT image and model. B: Gap of .9 mm clearly visible in 3D model. C: Gap of .5 mm not completely visible in 3D model. Note: the fracture next to the marked one is better visible due to the cortex discontinuity.


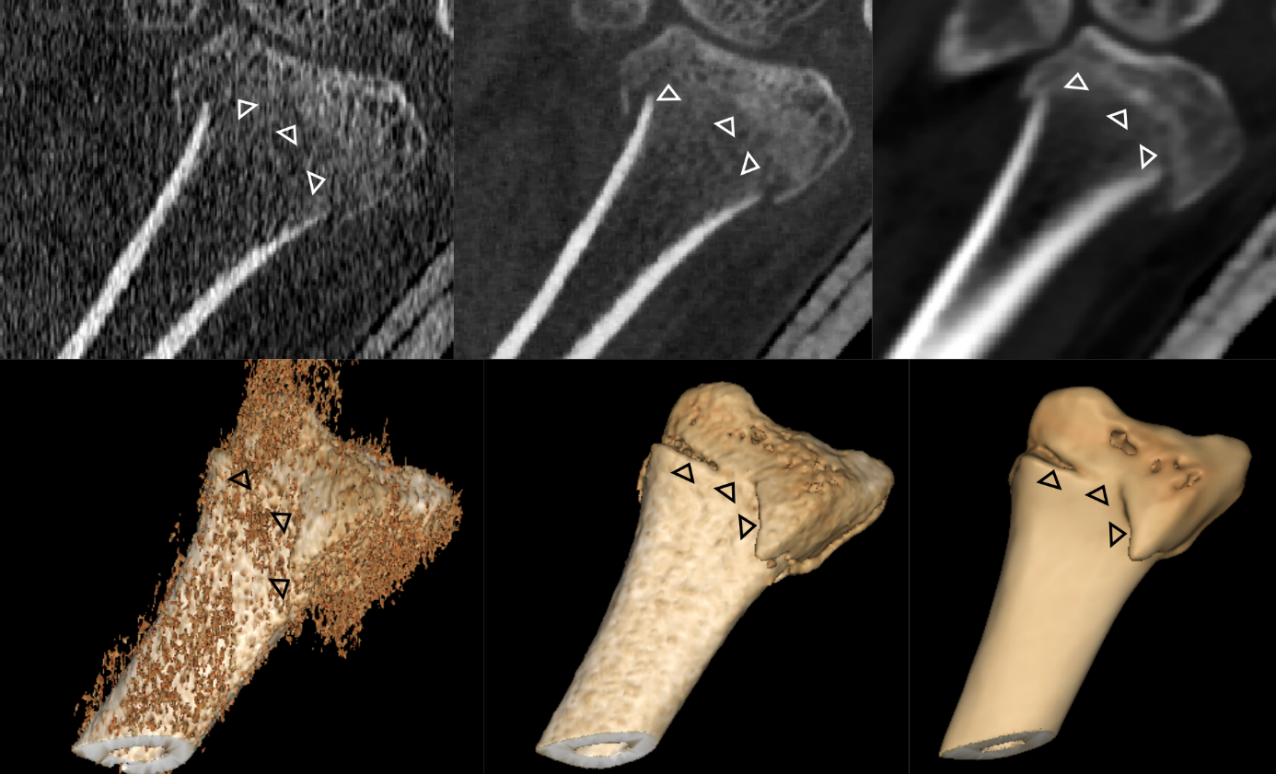


Figure S 6: Digital 3D models of a compression fracture of the distal radius (female, 42 years old) created from a CT imaging series (scanned at 120 kV, CT pixel size: .20 mm, slice thickness: .6 mm, increment: .3 mm, reconstruction kernel: Ur77u. Left & Middle: The original CT image series indicates a compression fracture with compression and displacement of the whole epi-metaphyseal region. The 3D model (left original, middle median filtered with 2-pixel window width) only indicates a fracture line of the ulnar epiphyseal bone and a displaced fragment of the radial part without displaying the slipping/compression of the whole epi-metaphyseal region. Right: CT image series reconstructed with Ur38u/3 kernel, demonstrating a thorough fracture line (with hyperdense region next to the fracture).


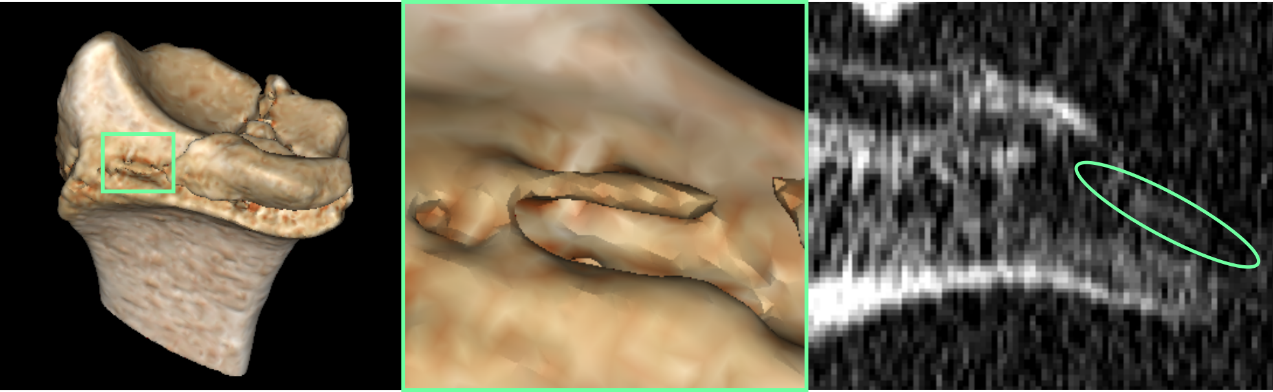


Figure S 7: Digital 3D model of a distal radius fracture with a minor displacement (male, 30 years old) created from a CT imaging series (scanned at 120 kV, CT pixel size: .31 mm, slice thickness: .6 mm, increment: .3 mm, reconstruction kernel: Ur77u). Left: 3D model. Middle: magnified 3D model showing bony displacement. C: CT image series with marked area of bony displacement (.5 mm thickness).

**Plain radiographs body donor specimens**

Plain radiographs were taken of body donor specimens in posteroanterior and lateral view with a standard protocol for the wrist (Fluorospot Compact, Siemens AG, Munich, Germany; operated at 51.8 kVp and 2.24 mAs (lateral view), and 49.9 kVp and 1.83 mAs (posteroanterior view), with a pixel spacing of .15 mm).

The detection rate of all bone lamellae was 100 %, as for incisions observed in the posteroanterior view. Incisions (200 and 400 µm) could not be determined reliably in the lateral view due to overlaying radius and ulna (see Figure S 8).

Intra- and inter-operator variability were determined in the same manner as done for CT image series and 3D models (see Table S 1 and Table S 2). In summary, Cohen’s kappa and Fleiss’ kappa were in the range of .66 to 1.00, indicating a substantial to almost perfect agreement. Hence, even fracture gaps and bony displacements of 100 µm can be reliably identified on standard clinical plain radiographs.

Figure S 8: Plain radiography for posteroanterior (left) and lateral (right) view. Insets show magnified areas highlighted in yellow.

Table S 1: Intra-operator variability (for operator 1) reported as Cohen’s kappa, adapted according to (1).

|  | **Lamellar width in µm** | | | | **Incision width in µm** | | **Fracture gap** | |
| --- | --- | --- | --- | --- | --- | --- | --- | --- |
|  | **400** | **300** | **200** | **100** | **400** | **200** | **Step** | **Gap** |
| X-ray dorsoventral | 1.00 | 1.00 | 1.00 | 1.00 | 1.00 | 1.00 | 1.00 | 1.00 |
| X-ray lateral | 1.00 | 1.00 | 1.00 | .80 | n/a | n/a | 1.00 | .88 |

Table S 2: Inter-operator variability (for all 5 operators) reported as Fleiss’ kappa, adapted according to (2).

|  | **Lamellar width in µm** | | | | **Incision width in µm** | | **Fracture gap** | |
| --- | --- | --- | --- | --- | --- | --- | --- | --- |
|  | **400** | **300** | **200** | **100** | **400** | **200** | **Step** | **Gap** |
| X-ray dorsoventral | 1.00 | 1.00 | 1.00 | .83 | 1.00 | 1.00 | .84 | 1.00 |
| X-ray lateral | .98 | .95 | .84 | .66 | n/a | n/a | .90 | .96 |

**REFERENCES**

1. Brennan RL, Prediger DJ. Coefficient Kappa: Some Uses, Misuses, and Alternatives. Educ Psychol Meas. 1981 Oct 1;41(3):687–99.

2. von Eye A. An Alternative to Cohen’s κ. Eur Psychol. 2006 Jan 1;11(1):12–24.
